# Tweets Classification on the Base of Sentiments for US Airline Companies

**DOI:** 10.3390/e21111078

**Published:** 2019-11-04

**Authors:** Furqan Rustam, Imran Ashraf, Arif Mehmood, Saleem Ullah, Gyu Sang Choi

**Affiliations:** 1 Department of Computer Science, Khwaja Fareed University of Engineering and Information Technology, Rahim Yar Khan, Punjab 64200, Pakistan; furqan.rustam1@gmail.com (F.R.); saleem.ullah@kfueit.edu.pk (S.U.); 2 Department of Information & Communication Engineering, Yeungnam University, Gyeongbuk 38541, Korea; ashrafimran@live.com

**Keywords:** text mining, text classification, sentiment analysis, supervised machine learning, ensemble classifier, long short-term memory network

## Abstract

The use of data from social networks such as Twitter has been increased during the last few years to improve political campaigns, quality of products and services, sentiment analysis, etc. Tweets classification based on user sentiments is a collaborative and important task for many organizations. This paper proposes a voting classifier (VC) to help sentiment analysis for such organizations. The VC is based on logistic regression (LR) and stochastic gradient descent classifier (SGDC) and uses a soft voting mechanism to make the final prediction. Tweets were classified into positive, negative and neutral classes based on the sentiments they contain. In addition, a variety of machine learning classifiers were evaluated using accuracy, precision, recall and F1 score as the performance metrics. The impact of feature extraction techniques, including term frequency (TF), term frequency-inverse document frequency (TF-IDF), and word2vec, on classification accuracy was investigated as well. Moreover, the performance of a deep long short-term memory (LSTM) network was analyzed on the selected dataset. The results show that the proposed VC performs better than that of other classifiers. The VC is able to achieve an accuracy of 0.789, and 0.791 with TF and TF-IDF feature extraction, respectively. The results demonstrate that ensemble classifiers achieve higher accuracy than non-ensemble classifiers. Experiments further proved that the performance of machine learning classifiers is better when TF-IDF is used as the feature extraction method. Word2vec feature extraction performs worse than TF and TF-IDF feature extraction. The LSTM achieves a lower accuracy than machine learning classifiers.

## 1. Introduction

Text mining is one of the distinguished fields of data mining which possesses the potential to extract useful information from raw data. In a world where 2.5 quintillion bytes of data are generated every day, text mining has become a key tool to retrieve meaningful data and organize them into profitable information [1,2]. Text classification is becoming a prominent field of research in text mining, especially after the inception and penetration of social platforms such as Facebook, Twitter, etc. People express their views on such platforms and their opinions serve as the guideline to design and govern the policies of various companies. For example, the tweets can be analyzed to find the sentiments of the users about a specific company or product, which helps to devise policies to increase the acceptance of products or improve user services. The wide use of such social platforms leads to generate many data that contain a variety of potential information.

The last few years have witnessed a growing interest in social network databases due to their richness and versatility. One iconic use of such data is to analyze user sentiments about a particular product or company. Such analysis of user sentiments from text is called “*sentiment analysis*’ [3]. Sentiment analysis is a famous method that is used to extract people’s reactions, opinions, reviews and feedback towards a specific product or service of a company. The user feedback on social platforms serves two broad purposes. First, the companies can model policies to attract new potential customers and revise the existing policies to increase the acceptance of their products/services based on sentiment analysis. For example, Rainie and Horrigan [4] pointed out that US presidential campaigns are planned according to the political reviews analyzed from Twitter data. In the same way, sentiment analysis is important for different companies to analyze customer reviews about products and make better decisions for the future [5,6]. Second, online reviews about various products and services have a significant influence on purchase trends [7]. Horrigan [8] pointed out that consumers are willing to pay more for a specific product which has a five-star rating than one that has a four-star rating.

**Figure 1:**
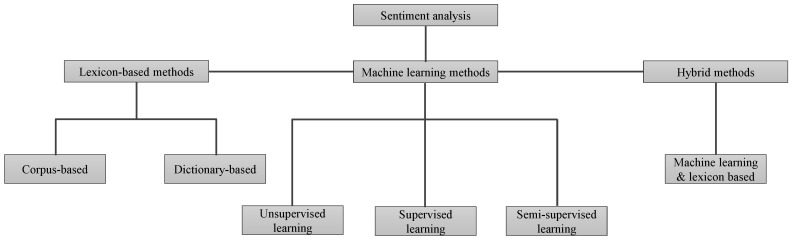
Categories of sentiment analysis.

Sentiment analysis can be divided into lexicon sentiment analysis technique, machine learning-based sentiment analysis, and hybrid methods [9], as shown in Figure 1. Lexicon sentiment analysis mainly works on the polarity of tokens (words) in a sentence. A lexicon is a dictionary or a container that contains a large set of standard words that are categorized based on the polarity score. However, most people use very informal words in reviews that are not present in lexicons. Therefore, researchers emphasize applying alternative techniques for sentiment detection in the text. Hence, the second category utilizes machine learning approaches for sentiment analysis. Models can be trained on a sample dataset and later can be used to perform predictions on a different dataset. The problem is formulated as a classification task, for example, a document can be represented by a set of features [5]. After that, these documents are labeled based on the polarity (i.e., positive, negative, or neutral), and converted into a feature matrix. In this way, machine learning approaches give better performance than that of lexicon-based method to detect sentiments [10].

The competition has been rising in every domain of life and airlines are no exception. They aim to generate more revenue by improving offered services and devising new schemes and policies for the future. Social networks play a very important role in such improvements, as the customer’s reviews serve as the feedback to such companies. Customers’ reviews are analyzed based on the expressions given in the reviews. The volume of such reviews is very high and it requires a large number of experts for analysis and classification. Thus, a variety of machine learning classifiers have been proposed which can help mitigate human effort to classify these reviews. However, improvements are still necessary to further increase the classification accuracy. This research proposes the use of a voting classifier to this end and aims to evaluate the performance of famous machine learning classifiers on a number of twitter datasets. This research serves the following key contributions:

- Machine learning-based classifiers including calibrated classifier (CC), support vector classifier (SVC), AdaBoost (ADB), decision tree classifier (DTC), Gaussian naive Bayes (GNB), extra trees classifier (ETC), random forest (RF), logistic regression (LR), stochastic gradient descent classifier (SGDC), and gradient boosting machine (GBM) are trained on US airline twitter dataset.
- A voting classifier (VC) is devised to perform tweets classification which is constituted by LR and SGDC.
- Complete and partial pre-processing schemes are adopted to evaluate the impact of pre-processing on models’ classification accuracy.
- Tweets are classified as positive, negative, or neutral and the results are compared against the actual classification to evaluate models’ performance.
- A deep learning long short-term memory (LSTM) network is implemented as well to analyze its performance on the selected dataset.

The rest of the paper is organized as follows. Section 2 describes a few pieces of research related to the current study. Section 3 gives an overview of the methodology adopted for the current research as well as a description of the dataset used for the experiment. Results are discussed in Section 4 while the conclusion is given in Section 5.

## 2. Literature Review

The area of text classification possesses a huge potential to analyze sentiments and many researchers have investigated the process of sentiment analysis by detecting emotions found in the text [11,12]. Others have proposed sentiment evaluation methods that are formulated by observing human responses to a certain experience [13]. The use of machine learning techniques including naïve Bayes (NB), maximum entropy (ME), and support vector machines (SVM) for sentiment classification has also been studied [14]. For example, the authors of [15] applied NB, ME, and SVM on the Internet Movie Database (IMDb), which consists of movie reviews expressed either with stars or in numerical values. The approach is evaluated using accuracy and recall measures. This work has served as a baseline for many authors and the same techniques have been utilized across different domains.

Similarly, the authors of [16] performed sentiment analysis on travelers’ feedback about airlines. The authors found that the feature selection and over-sampling techniques are equally important to achieve refined results. Feature analysis is performed to select the best features which not only improves the overall performance of the model but reduces the training time as well. In addition, the skewed distribution of the classes found in most of the smaller datasets is reduced without causing over-fitting. The results of the research show the compelling evidence that the proposed model has a higher classification accuracy when predicting the three classes of positive, negative, and neutral. The authors of [17] followed a similar approach and performed a multi-class sentiment classification. A feature selection process is used to extract the important features that are later used to train a machine learning-based algorithm. The performance of DTC, NB, SVM, radial basis function neural network, and k nearest neighbor is tested with 10-fold cross-validation.

In another research [18], the authors used customers feedback to investigate different aspects such as loyalty, satisfaction, etc. The loyalty is determined through airline attributes, namely operational factors (punctuality, aircraft, and safety), attractive factors (food and beverages and the staff service), competitive factors (schedule, ticket prices, reputation, and flyer program), etc. The research concludes that the customer’s higher satisfaction can be achieved through company reputation, staff service, frequent flyer program, aircraft, and punctuality. Kumar and Sebastian [19] presented a novel approach for the sentiment analysis of Twitter data. To uncover the sentiment, the authors extracted the opinion words (a combination of the adjectives along with the verbs and adverbs) in the tweets. The corpus-based method is used to find the semantic orientation of adjectives and the dictionary-based method to find the semantic orientation of verbs and adverbs. The overall tweet sentiment is then calculated using a linear equation that also incorporates emotion intensifiers. A score is calculated for the overall sentiment of the tweet and tweets are classified as positive, neutral and negative based on the calculated score.

The authors of [20] performed sentiment analysis using a machine learning technique. The polarity is found using TextBlob, SentiWordNet and word sense disambiguation (WSD) sentiment analyzers. TextBlob comes with the basic features of natural-language processing essentials, which are used for the polarity and subjectivity calculation of tweets. SentiWordNet is a publicly available analyzer for the English language that contains opinions extracted from a wordnet database. In addition, W-WSD has the ability to detect the correct word sense within a specified context.

The authors of [21] presented a meta-heuristic method called CSK, which is based on cuckoo search (CS) and k-means (K). Since clustering plays a vital role in analyzing the viewpoints and sentiments in user tweets, the research proposes a method that is used to find the optimum cluster head from the twitter dataset. Experimental results show promising outcomes. The authors of [22] investigated the impact of multiple classifier systems on Turkish sentiment classification. The voting algorithm is used with NB, SVM, and bagging to evaluate their efficacy. The results demonstrate that the use of multiple classifiers elevates the performance of individual classifiers. The research approves that multiple classifier systems have more potential for sentiment classification.

In addition to the use of multiple classifiers for classification, employing various pre-processing techniques helps to improve the classification as well. For example, the authors of [23] proved that the selection of an appropriate pre-processing technique may produce enhanced classification performance. They investigated a variety of pre-processing techniques including term weighting, frequency cut, stemming, and stopword elimination to analyze their impact on machine learning-based classification methods. Their research shows that the combination of various pre-processing methods plays a decisive role in finding the best classification rates. They also studied the pre-processing techniques and their relevant impact on the feature space through visualization.

In the same fashion, the use of various feature extraction techniques has proven to improve classification accuracy. Text mining has many feature extraction methods but term frequency (TF), inverse document frequency (IDF), TF-IDF, word2vec and doc2vec are among the most commonly used feature extraction techniques [24]. The authors of [25] investigated the use of TF, IDF, and TF-IDF with linear classifiers including SVM, LR, and perceptron with a native language identification system. Experiments are carried out with ten-fold cross-validation on different languages. The TF-IDF is applied to n-gram words/characters/ parts-of-speech tags. The TF-IDF weighting on features proves to outperform other techniques when applied with uni-grams and bi-grams of words. Similarly, the authors of [26] analyzed the use of three feature extraction techniques with a neural network for the text analysis. TF-IDF along with its two modifications, namely latent semantic analysis (LSA) and linear discriminant analysis (LDA), is applied to evaluate the performance of each feature analysis technique. The experiment shows that TF-IDF helps the model to achieve higher accuracy with large dataset. For smaller datasets, the combination of TF-IDF and LSA is appropriate to achieve similar accuracy.

Machine learning techniques perform better for classification than that of traditional approaches. However, machine learning methods for classification problems commonly assume that the class values are unordered. However, in many practical applications, the class values exhibit a natural order, for example, when learning how to grade. The standard ordinal classification approach converts the class value into a numeric quantity, applies a regression learner to the transformed data and translates the output back into a discrete class value in a post-processing step. The authors of [27] presented a simple method that enables standard classification algorithms to make use of ordering information in class attributes. Tree induction methods and linear models are popular techniques for supervised learning tasks, both for the prediction of nominal classes and numeric values. For predicting numeric quantities, research has been conducted on combining two schemes into “model trees”, i.e., trees that contain linear regression functions at the leaves. The authors of [28] presented an algorithm that performs classification using logistic regression instead of linear regression. A stage-wise fitting process is used to construct the logistic regression models that can select relevant attributes in the data in a natural way and shows how this approach can be used to build the logistic regression models at the leaves by incrementally refining those constructed at higher levels in the tree. In the current research, supervised learning algorithms are used, wherein some algorithms perform individually while others use ensemble learning techniques.

## 3. Materials and Methods

This section contains the description of the dataset used for sentiment analysis, its visualization, as well as the proposed methodology to perform the sentiment analysis on the selected dataset.

### 3.1. Data Description

In this study, the dataset from Kaggle was used, which contains tweets for six airlines of the United States (US). The dataset name is “twitter-airline-sentiment” and it contains a total of 14,640 records. Every record is labeled as positive, negative, or neutral according to the sentiment polarity. The selected dataset contains different features and its description is given in Table 1.

### 3.2. Data Visualization

**Figure 2:**
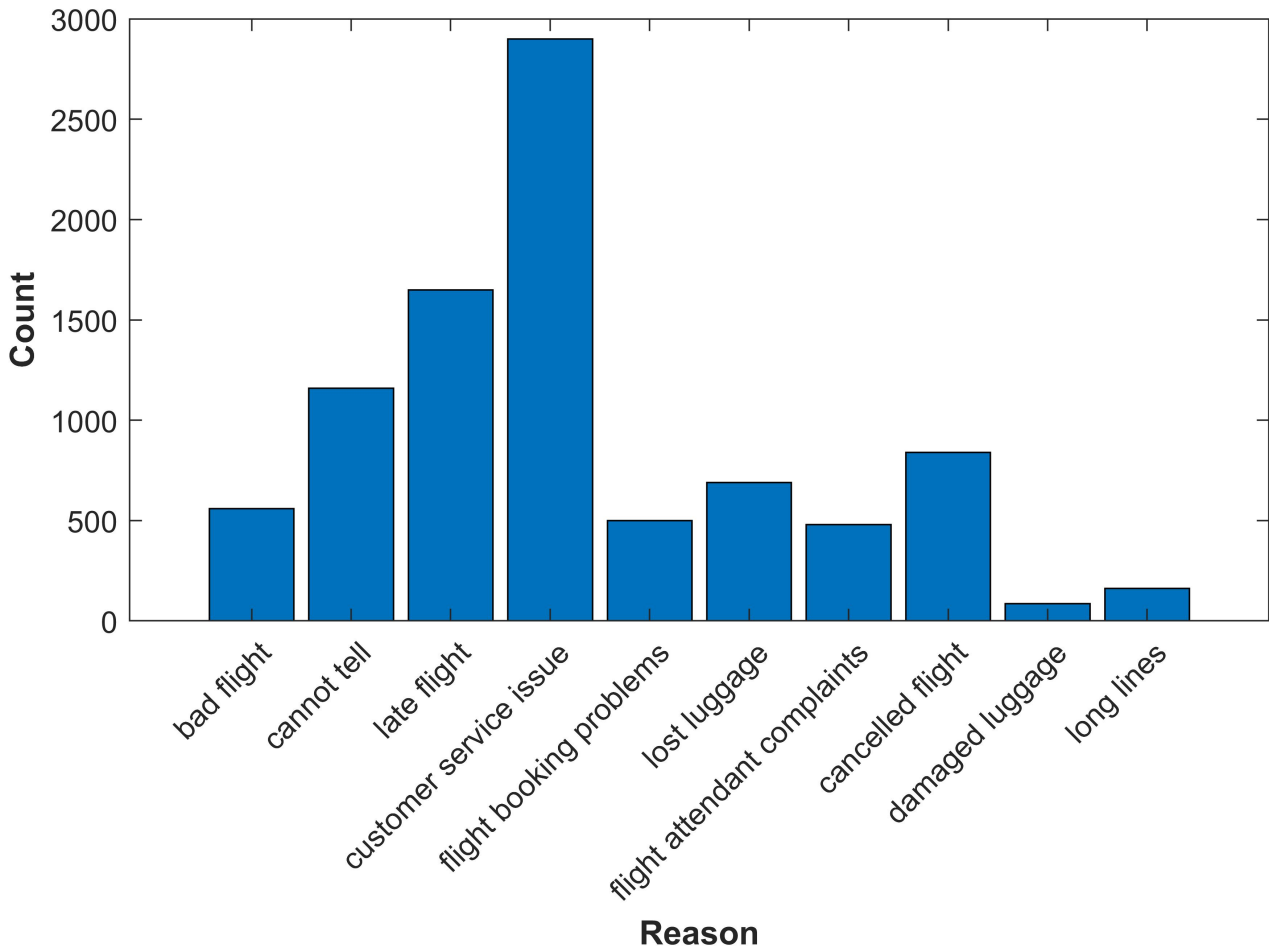
Customers’ complaints of airlines.

**Figure 3:**
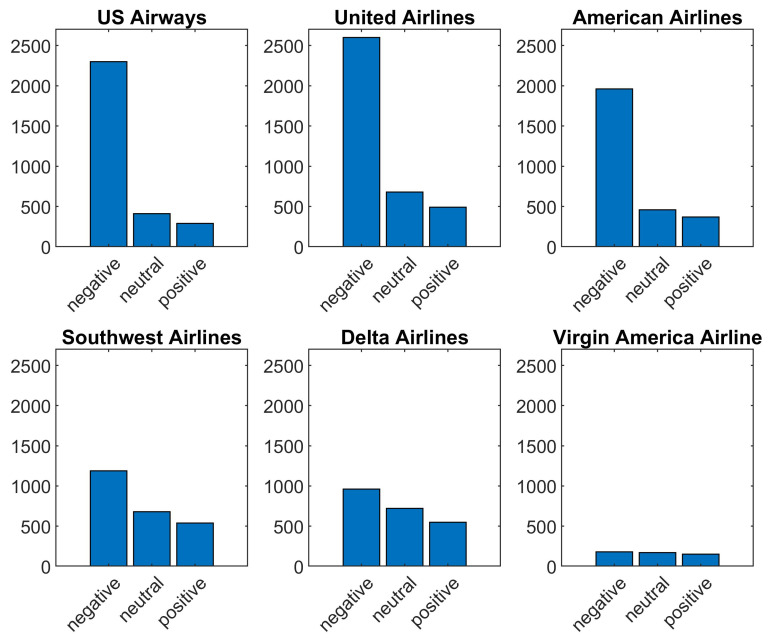
The polarity of customers’ tweets for individual airline.

The dataset is visualized to help understand its attributes. Figure 2 shows the most frequent reasons for customer complaints about the airline. The dataset visualization shows that the highest number of tweets are about “customer service issues”. Figure 3 shows sentiment polarity for six airlines used as the standard to evaluate the performance of the selected classifiers.

### 3.3. Methodology

In the current research, different classifiers and feature extraction techniques were investigated. CC, LR, ETC, SVC, DTC, RF, ADB, GNB, SGDC, and GBM were evaluated on the selected dataset with term frequency (TF), term frequency-inverse document frequency (TF-IDF) and word2vec features. The phases followed during the experiments are described here briefly.

In the methodology steps of this research, pre-processing was carried out on the dataset. Different tools and libraries were utilized in this step, e.g., natural language toolkit. This study considered two strategies at the pre-processing level:

- Complete pre-processing
- Partial pre-processing

#### 3.3.1. Complete Pre-processing

In complete pre-processing, data cleaning was performed to improve the learning efficiency of machine learning models. Machine learning models show improved classification accuracy if the data are pre-processed. The pre-processing was done using the natural language toolkit of Python [29]. Tweets contain punctuation, stopwords, and the combination of lower- and uppercase words, which can affect the model learning capability. Two tweets are shown in Table 2 as a means to show the pre-processing steps followed in this study.

**Figure 4:**
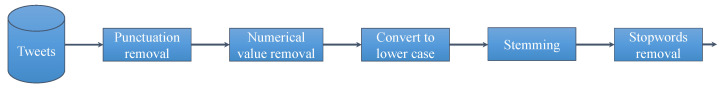
The sequence followed in pre-processing of tweets dataset.

Figure 4 shows the sequence of the pr-processing steps followed for the selected twitter dataset. As a first step, punctuation has to be removed from tweets. The following punctuation was removed from text: []() \/ | , ; . ’. In addition, twitter assigned @user to each user was also removed during this phase. Table 3 shows the tweets before and after punctuation removal.

Punctuation was removed from data because it does not contribute to text analysis in the study. Punctuation helps to make sentences readable but it impairs the models’ ability to differentiate between punctuation and other characters [30]. In the next step, numeric values from the tweets were removed as they have no impact on text analysis. Removing numeric values decreases the complexity of training the models. Table 4 shows the output of before and after numbers were removed from tweets.

After numeric removal, all text in the tweets was converted to lowercase. This step is important because text analysis is case sensitive. Yang and Zhang [30] stated that the probabilistic machine learning models count the occurrence of each word, which means that, e.g., “Good”, and “good” are considered two different words if changing all text to lowercase is not performed. It could decrease the importance of more frequent terms in the text. Table 5 shows the example of before and after the tweets have been converted to lower case.

Stemming is an important technique in pre-processing because removing affixes from words and converting them into their root form helps to increase the performance of the model [31]. For example, words may have many forms with essentially the same meaning in the text. For example, “goes” and “going” are modified forms of “go”. Stemming converts these types words into their root form. Stemming was performed using Porter stemmer algorithms in current study [32]. Table 6 shows the sample of tweets before and after stemming.

The last step in the pre-processing phase is the removal of stopwords in the tweets. Stopwords have no analytic value for text analysis, so they need to be removed to reduce the complexity of input features. Table 7 shows the output of the tweets after stopwords were removed.

#### 3.3.2. Partial Pre-processing

**Figure 5:**
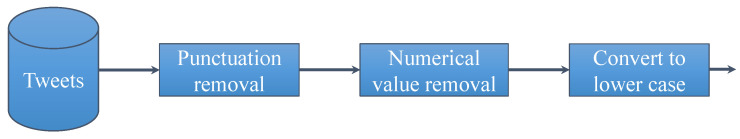
Steps followed in partial pre-processing.

Other than the complete pre-processing, this study considered the use of partial pre-processing as well, to analyze the impact of pre-processing steps on classifiers’ accuracy. The partial pre-processing does not involve “stemming” and “stopwords removal”. Thus, the pre-processing was carried out in the order given in Figure 5.

#### 3.3.3. Feature Extraction Methods

**Figure 6:**
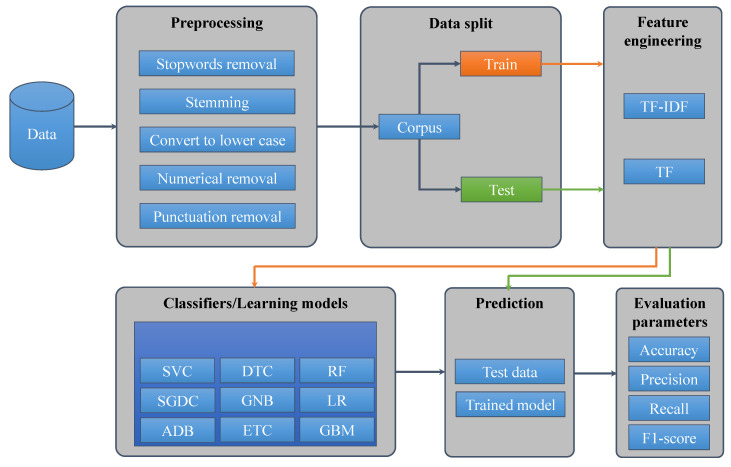
The methodology adopted for tweets classification.

After the pre-processing phase, the corpus was divided into “training subset” and “testing subset”. It was divided in the ratio of 3:1 for training and testing, respectively. Feature extraction methods were then applied to the training subset, as shown in Figure 6, which represents the adopted methodology.

Feature extraction techniques were applied to both training and testing data: on the training data to train the selected models and on the testing data when classification was performed. TF-IDF is a scoring measure widely used in information retrieval (IR) and summarization. TF-IDF is intended to reflect how relevant a term is in a given document. TF-IDF feature extraction considers TF and IDF. IDF rewards the tokens that are rare overall in a dataset. If a rare word appears in two documents, then it is more important to the meaning of each document. IDF weights a token *t* in a set of documents *U* and is computed as follows:

(1) $$IDF\left(t\right)=\frac{N}{n(t)}$$

where $\frac{n(t)}{N}$ is the frequency of *t* in *U* and $\frac{N}{n(t)}$ is the inverse frequency. Thus, the total TF-IDF weight for a token in a document is given as:

(2) TF−IDF=TF∗IDF

TF-IDF is used with parameter “ngram_ range”. TF-IDF is used to measure the importance weight of terms which give the weights of each term in the corpus. The term weighted matrix is the output of TF-IDF. With the TF-IDF vectorizer, the value increases proportionally to the count but is offset by the frequency of the word in the corpus. Table 8 shows the output of three sentences when TF-IDF technique is applied to the pre-processed form of these sentences. The sentences are:

“good companies”

“bad services”

“I have seen good management”

Similar to TF-IDF, the TF technique is used for feature extraction as well and is commonly applied in document classification where the (frequency) occurrence of each word is used as a feature for training a classifier. However, contrary to TF-IDF where more frequent words get smaller weight, the TF feature does not care if a word is common or not. The output of TF for the above-given sentences is shown in Table 9.

This study also considers the use of word2vec as the feature extraction technique [33]. Word2vec is a famous two-layer neural net which produces the feature vectors from a text corpus. It utilizes the continuous bag-of-words (CBOW) or the skip-gram (SG) model for this purpose. This study employed SG because SG has been tested and shown good performance in NLP tasks [34,35]. The use of SG model aims at finding the word representations that are used to predict the adjacent word in a sentence. The SG model was considered in this study based on its suitability for small- to medium-sized datasets. Jang et al. [36] stated that the SG model is advantageous over CBOW when data size is not too large.

### 3.4. Classifiers Used for Tweet Classification

This section describes the necessary details for the machine learning classifiers used in this study for tweet classification.

#### 3.4.1. Machine Learning Classifiers

Multiple classifiers were used in the current study. The DTC is one of the used classifiers. The DTC algorithm falls under the category of supervised learning and can be used to solve both regression and classification problems. In DTC, the major challenge is the identification of the attribute for the root node at each level [37]. This process is known as attribute selection. The two most popular attribute selection measures are “information gain” and “Gini index” [38]. To calculate Gini, this study considered the probability of finding each class after a node and then the sum of the square of those values was calculated and subtracted from 1. Thus, when a subset is pure (i.e., there is only one class in it), Gini will be 0, because the probability of finding that class is 1; indeed, it is concluded that we have reached a leaf. To calculate Gini value, the following equation is used:

(3) $$Gini=1-\sum_{i=1}^{classes}p{\left(i|t\right)}^{2}$$

Besides Gini, information gain was also used for the selection of the best attribute. Whereas the Gini value gives the impurity of data in the dataset, information gain provides the purity of data in the dataset. There are two steps for calculating information gain for each attribute:

Step 1. Calculate the entropy of the target.

Step 2. Calculate the entropy for every attribute.

Using information gain formula, entropy was subtracted from the entropy of target. Given a set of examples *D*, entropy is calculated using:

(4) $$entropy\left(D\right)=-\sum_{i=1}^{\left|c\right|}P_{r}\left(c_{i}\right)\log_{2}P_{r}\left(c_{i}\right),$$

(5) $$\sum_{i=1}^{\left|c\right|}P_{r}\left(c_{i}\right)=1$$

where $P_{r}\left(c_{i}\right)$ is the probability of class $c_{i}$ in dataset *D*.

The entropy is used as the measure of impurity or disorder of a dataset (or a measure of information in a tree). If an attribute $A_{i}$ is made with *v* values, this will partition *D* into *v* subsets $D_{1},D_{2},...,D_{v}$. If $A_{i}$ is used as the current root, the expected entropy is:

(6) $$entropy_{A_{i}}\left(D\right)=-\sum_{j=1}^{v}\frac{|D_{j}|}{D}*entropy\left(D_{j}\right)$$

Thus, information gain for selecting attribute $A_{i}$ to branch or partition the data is:

(7) $$entropy\left(D,A_{i}\right)=entropy\left(D\right)-entropy_{A_{i}}\left(D\right)$$

The attribute with the highest gain was selected to branch/split the current tree in this study. The Gini value and information gain were used to construct the trees for all tree-based classifiers used in this study.

SVM is another machine learning classifier utilized in the current study. It is a linear model for classification and regression problems. It can solve linear and non-linear problems and works well for many practical applications [39]. SVM creates a line or a hyper-plane which separates the data into classes. SVM has functions called kernels which take low-dimensional input space and transform it into a higher-dimensional space, i.e., it converts not separable problems to separable problems. It is mostly useful in non-linear separation problems. Simply put, it does some extremely complex data transformations and finds the process to separate the data based on the defined labels.

Two voting classifiers, namely LR and SGDC, were evaluated as well. Both LR and SGDC are able to estimate class probabilities on their outputs, i.e., they predict if the input is class-*A* with probability *a* and class-*B* with probability *b*. If a>b, then it outputs predicted class is *A*, otherwise *B*. In voting, the classifier sets the voting parameter to soft enable them in order to calculate their probability (also known as confidence score) individually and presents it to the voting classifier. Then, the voting classifier averages them and outputs the class with the highest probability. The GBM, on the other hand, trains many models in a gradual, additive and sequential manner. The major difference between ADB and GBM is how the two algorithms identify the shortcomings of weak learners (e.g., decision trees). The ADB model identifies the shortcomings by using high weight data points, while the GBM model performs the same by using gradients in the loss function y=ax+b+e, where *e* is the error term. The loss function is a measure indicating how good the model’s coefficients are at fitting the underlying data. A logical understanding of loss function would depend on what we are trying to optimize.

#### 3.4.2. Proposed Voting Classifier (LR + SGDC)

**Figure 7:**
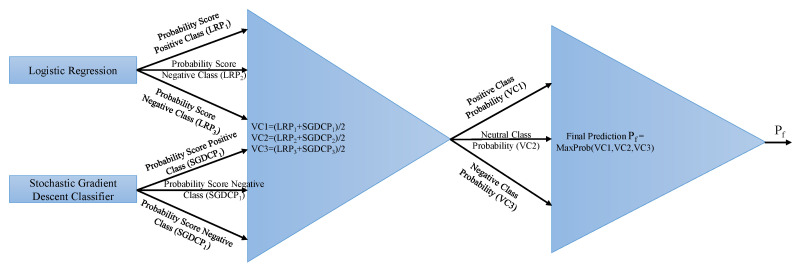
Architecture of the proposed voting classifier.

The voting classifier (VC) is an ensemble model that combines different base models to perform the classification through different voting schemes (e.g., soft voting and hard voting). It gets final results by aggregating the results from the classifiers. In this study, two classifiers, LR and SGDC, were ensembled through soft voting criteria for the final prediction of target class. SGDC is useful for big data, especially when there are redundancies in the dataset. It solves the classification problems by specifying a loss and penalty function [40]. It works similarly to regular gradient descent, except that it looks at only one sample at each step [41]. LR, on the other hand, derives the posterior class probability (PCP) p(Ct|v) implicitly to perform the binary classification. LR derives PCP through the sigmoid function σ by using a linear combination of the input [42]. VC can be expressed as:

(8) $$\hat{p}=argmax\left\{\sum_{i}^{n}LR_{i},\sum_{i}^{n}SGDC_{i}\right\}$$

where $\sum_{i}^{n}LR_{i}$ and $\sum_{i}^{n}SGDC_{i}$ give *n* prediction probabilities for given samples. After each given probability for sample text, the probability passes through soft voting criteria, as shown in Figure 7.

The functioning of the VC can be described with the help of an example. Let the following be the probability scores of each class given by LR:

Negative class = 0.1126337

Neutral class = 0.35984473

Positive class = 0.52889191

Similarly, SGDC probability scores against each class are:

Negative class = 0.17610406

Neutral class = 0.42969437

Positive class = 0.39420157

The VC gives probability scores against each class using the probabilities of LR and SGDC as follows:

Negative class = (0.1126337+0.17610406)/2 = 0.143683715

Neutral class = (0.35984473 + 0.42969437)/2 = 0.39476955

Positive class = (0.52889191 + 0.39420157)/2 = 0.46154674

The VC classifies it as the “positive” class for the given tweet using the maximum of the given probability. The tweet tested with the VC classifier also belongs to the “positive” class in the dataset.

### 3.5. Performance Evaluations Parameters

Different performance evaluation parameters have been utilized to analyze the performance of the classifiers. Four basic notations used in these parameters are as follows [43,44,45]:

**True Positives (TP):** These are the positive predictions of a class made by a classifier which are correctly predicted.

**True Negatives (TN):** These are the negative predictions about a class which are correctly labeled so by the classifier.

**False Positives (FP):** These are the negative instances of a class which are incorrectly predicted as positive by the classifier.

**False Negatives (FN):** These are the positive instances of a class which are incorrectly predicted as negative by the classifier.

These quantities are used to calculate accuracy, F1 score, recall, and precision of each classifier to evaluate its performance. Accuracy is defined as:

(9) $$accuracy=\frac{TP+TN}{TP+TN+FP+FN}\times 100$$

Recall shows the completeness of a classifier and is calculated as:

(10) $$Recall=\frac{TP}{TP+FN}$$

Precision is the exactness of the classifiers and involves TP to the sum of TP and FP. It is calculated using:

(11) $$Precision=\frac{TP}{TP+FP}$$

The F1 score conveys the balance between the precision and the recall and is calculated as:

(12) $$F_{1}=2\frac{precision\times recall}{precison+recall}$$

## 4. Results and Discussion

Experiment results are discussed with respect to various pre-processing steps utilized as well as the feature extraction techniques selected for this study.

### 4.1. Results with Complete Pre-processing

The current research utilized the selected machine learning classifiers with different hyper-parameters. These parameters were set empirically to achieve higher accuracy. CC, for example, performs best when it works with stochastic gradient descent. Similarly, the SVM classifier gives higher accuracy with a linear kernel. The accuracy results for all classifiers when used TF-IDF are displayed in Table 10.

**Figure 8:**
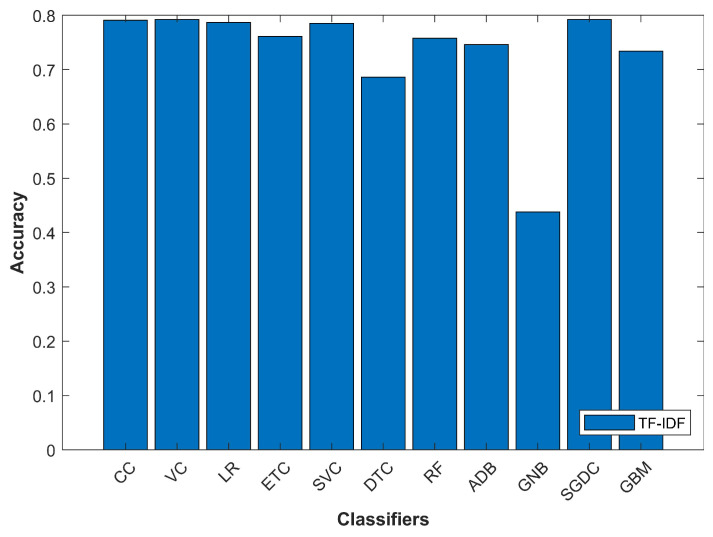
Classifiers’ accuracy with TF-IDF.

As shown in Figure 8, GNB gives the lowest accuracy when used with TF-IDF feature extraction technique and VC gives the best results with TF-IDF. VC gives the best results as it is an ensemble model that works with other classifiers.

Table 11 shows the classification accuracy of different classifiers when used with TF feature extraction method. Experimental results reveal that the SGDC classifier shows the best results when stopping criterion parameter value is set to “1e${}^{-3}$” and max_ iter=1000. Similarly, VC gives higher accuracy than that of other classifiers when it works with LR and SGDC.

**Figure 9:**
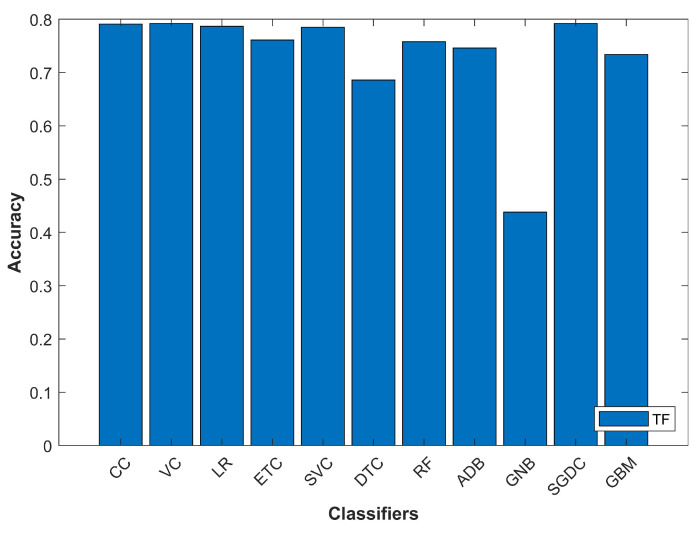
Classifiers’ accuracy with TF feature extraction.

Figure 9 shows that SGDC performs better than other classifiers, even when the TF feature extraction method is utilized. There is a slight difference in the accuracy of RF on TF-IDF and TF techniques. However, at the same time, VC shows a very similar performance with both feature extraction techniques.

Table 12 shows the results for precision, recall, and F1 score of each class of tweets, as well as the average of three classes for all classifiers used in the study. In the current study, CC performs better when used with TF-IDF because it allows the calibration of the probabilities for a given model or to add support for probability prediction on the dataset. VC is the best classifier for tweets classification on the selected dataset when the TF-IDF feature extraction method is used. The average precision of VC is 82%, which is better than CC.

LR examines the influence of various factors on a dichotomous outcome by estimating the probability of the event’s occurrence [46]. It gives good results on the dataset with TF-IDF. The precision of LR is better on neutral and positive labels than VC. Additionally, ETC, RF, SVM, and SGDC perform better than the other classifiers in terms of precision, recall and F1 score. SVM shows better results with linear kernel and parameter *c* set to 2.0. DTC, GBM, ADB, and GNB do not perform well and have lower precision on the selected dataset. Table 12 shows the performance evaluation parameters for the selected classifiers with TF-IDF feature extraction method.

The results for performance parameters when selected classifiers make use of the TF feature extraction method are given in Table 13. The results demonstrate that CC, VC, LR, ETC, and RF perform better with both TF and TF-IDF feature extraction methods. On the other hand, the performance of GNB is severely degraded when used with TF feature extraction.

Table 14 shows the accuracy of selected classifiers when word2vec is used as the feature extraction method. The results show that the performance of all classifiers has been degraded with the exception of SVC and GNB. The accuracy of SVC has been slightly improved, while GNB’s accuracy has been significantly elevated with word2vec features. Table 15 shows the comparison of classifiers’ accuracy when used with various feature extraction methods. The results show that the accuracy of most classifiers is degraded with word2vec features. CC is able to achieve the highest accuracy of 0.780 with word2vec while many classifiers have a substantial decrease in accuracy.

**Figure 10:**
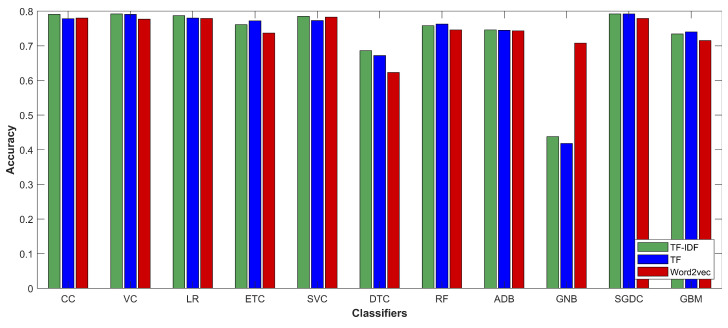
Comparison of classifiers’ accuracy with TF and TF-IDF feature extraction.

The average accuracy of all the classifiers with TF and TF-IDF is shown in Figure 10. The experimental results show that there is very little difference in accuracy when the feature extraction technique is changed from TF to TF-IDF; however, TF-IDF is better in terms of accuracy, precision, and other performance metrics.

### 4.2. Results with Partial Pre-Processing

Table 16 compares the results for classifiers’ accuracy using complete pre-processing with the partial pre-processing results where stopwords removal and stemming has been discarded. The results exhibit that the partial pre-processing leads to reducing the overall accuracy of the classifiers. Complete pre-processing is of utmost importance to improve the performance of prediction. Stemming and stopwords removal help to mitigate the amount of meaningless data and reduce the data dimensionality [47]. Document features estimate the importance of specific terms in a document. Pre-processing helps to reduce the high-dimensional attributes and help feature extraction methods to learn only the important features. Hence, if the pre-processing is incomplete, the feature extraction may be improper, which leads to poor prediction of classifiers.

### 4.3. Results Using Long Short-Term Memory Classifier

**Figure 11:**
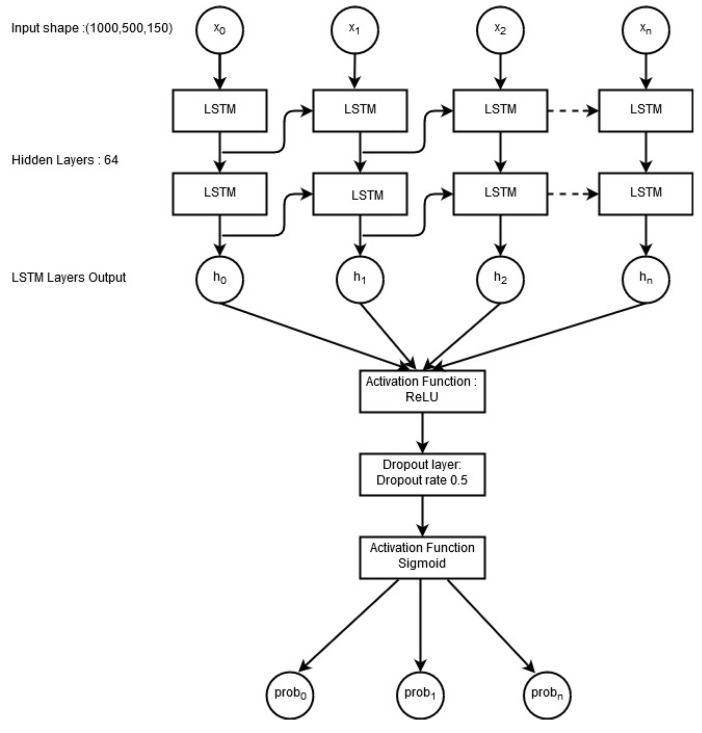
Architecture of LSTM model used in this study.

A deep learning approach LSTM was employed as well to analyze its accuracy on the selected twitter dataset. Figure 11 shows the structure of the LSTM network used in this study.

Between the input layer and LSTM layer, an embedding layer was inserted, which creates word vectors from the input layer. Rectified linear unit (ReLU) was used as the activation function because of its better performance on text data [41,48]. A dropout layer was used as the regularization unit with a value of 0.5. Sigmoid was utilized at the end layer to produce the probability of each class [49]. This study used “Adam” optimizer as it has proven to show better performance in case of noisy data [50]. The LSTM is able to achieve an accuracy of 0.686, which is lower than those of most of the machine learning models investigated in this study. The poor performance of LSTM is due to the dataset used in the current study. Deep learning is a data-intensive approach that performs better when the dataset is large. Research shows various results both favorable and poor on the use of deep learning on smaller datasets. For example, [51] stated that deep learning-based methods perform poor with small datasets. However, Zampieri et al. [52] investigated the use of SVM, CNN, and bidirectional LSTM (BiLSTM) to predict the offensive language in social media. They found that BiLSTM and CNN can perform well even on relatively smaller datasets, and outperform traditional machine learning SVM. It is also possible that the specific architecture of the used LSTM model is not suitable for the selected dataset. Hence, the results cannot be conclusive without further investigation of LSTM and other deep learning techniques on more datasets.

### 4.4. Statistical Significance of Results

This study employed a T-test to validate if the difference between the results is statistically significant or not. Thus, the null hypothesis $H_{o}$ is that the difference between the classification accuracy is not significant while the alternative hypothesis $H_{a}$ claims that the difference between the accuracy is significant. The T-test found that the null hypothesis cannot be rejected in favor of alternative hypotheses for TF and TF-IDF features. However, when the test was performed between the accuracy with TF-IDF and word2vec features, it favored the alternative hypothesis and stated that the difference in the accuracy is statistically significant.

## 5. Conclusions

This paper proposes a voting classifier that is based on logistic regression and stochastic gradient descent classifier. Soft voting is used to combine the probability of LR and SGDC. In addition, various machine learning-based text classification methods were investigated to perform sentiment analysis. The experiments were carried out on a twitter dataset which contains the reviews of travelers about US airlines. Three feature extraction methods, namely TF, TF-IDF, and word2vec, were investigated to analyze the impact on models’ classification accuracy. The selected classifiers were used to classify the tweets into positive, negative and neutral classes. Precision, recall, and F1 score were used as performance metrics besides accuracy. The results demonstrate that TF-IDF feature extraction is more appropriate for tweet classification. The proposed voting classifier performs better with both feature extraction methods and achieves an accuracy of 0.789 and 0.791 with TF and TF-IDF, respectively. Ensemble classifiers show higher accuracy than the non-ensemble classifiers. A deep long short-term memory model was also implemented with TF-IDF feature extraction. The results show that LSTM does not perform well on the selected dataset. However, the results from LSTM are not conclusive, as research [52] shows strong evidence of superiority of bidirectional LSTM and CNN over machine learning classifiers. Thus, future work is intended to perform further experiments with more deep learning methods on the selected as well as additional datasets.

## Figures and Tables

**Table 1:** Feature description of selected dataset.

Featuresr	Descriptiont
Airline Sentiment Confidence	A numeric feature representing the confidence level of classifying the tweet to one of the 3 classes.
Negative Reason	The reason behind considering this tweet as negative.
Negative Reason Confidence	The level of confidence in determining the negative reason behind a negative tweet.
Airline	Name of the airline Company.
Retweet Count	Number of retweets of a tweet.
Text	Original tweet posted by the user.
Airline Sentiment	Labels for tweets (positive, negative, neutral).

**Table 2:** Sample tweets from twitter dataset.

No.	Tweets
1	@VirginAmerica plus you’ve added commercials to the experience... tacky.
2	@VirginAmerica I didn’t today... Must mean i need to take another trip for 2 months!

**Table 3:** Output of sample after removing punctuation.

Input Data	After Punctuation Removal
@VirginAmerica plus you’ve added commercials to the experience... tacky.	plus youve added commercials to the experience tack
@VirginAmerica I didn’t today... Must mean i need to take another trip for 2 months!	I didnt today Must mean i need to take another trip for 2 months

**Table 4:** Output of sample after numbers removal.

Input Data	After Numeric Removal
plus youve added commercials to the experience tacky	plus youve added commercials to the experience tacky
I didnt today Must mean i need to take another trip for 2 months	I didnt today Must mean i need to take another trip for months

**Table 5:** Output of sample after case lowering of tweets.

Input Data	After Case Lowering
plus youve added commercials to the experience tacky	plus youve add commercials to the experience tacki
I didnt today Must mean i need to take another trip for months	i didnt today must mean i need to take another trip for months

**Table 6:** Tweets before and after stemming.

Input Data	After Stemming
plus youve added commercials to the experience tacky	plus youve add commercial to the experience tacki
i didnt today must mean i need to take another trip for months	i didnt today must mean i need to take another trip for month

**Table 7:** Output of tweets after stopwords removal.

Input Data	After Stopwords Removal
plus youve add commercial to the experience tacki	plus haveve add commercial experience tacki
i didnt today must mean i need to take another trip for month	today must mean need take another trip month

**Table 8:** Output of TF-IDF on preprocessed data.

Bad	Compani	Good	Management	Seen	Service
0.000000	0. 795961	0. 605349	0. 000000	0. 000000	0.000000
0.707107	0. 000000	0. 000000	0. 000000	0.000000	0.707107
0. 000000	0. 000000	0.473630	0.622766	0.622766	0.000000

**Table 9:** Output of TF on preprocessed data.

Bad	Compani	Good	Management	Seen	Service
0	1	1	0	0	0
1	0	0	0	0	1
0	0	1	1	1	0

**Table 10:** Accuracy of models with TF-IDF.

Classifier	Features Used	Accuracy
AdaBoost Classifier	TF-IDF	0.746
Calibrated Classifier	TF-IDF	0.791
Decision Tree Classifier	TF-IDF	0.686
Extra Trees Classifier	TF-IDF	0.761
Gaussian Naïve Bayes	TF-IDF	0.438
Gradient Boosting Machine	TF-IDF	0.734
Logistic Regression	TF-IDF	0.787
Random Forest Classifier	TF-IDF	0.758
Stochastic Gradient Descent classifier	TF-IDF	0.792
Support Vector Classifier	TF-IDF	0.785
Voting Classifier (LR + SGDC)	TF-IDF	0.792

**Table 11:** Classifiers’ accuracy with TF feature extraction.

Classifier	Features Used	Accuracy
AdaBoost Classifier	TF	0.745
Calibrated Classifier	TF	0.789
Decision Tree Classifier	TF	0.672
Extra Trees Classifier	TF	0.772
Gaussian Naïve Bayes	TF	0.418
Gradient Boosting Machine	TF	0.740
Logistic Regression	TF	0.780
Random Forest Classifier	TF	0.763
Stochastic Gradient Descent classifier	TF	0.792
Support Vector Classifier	TF	0.773
Voting Classifier (LR + SGDC)	TF	0.791

**Table 12:** Classifiers’ accuracy parameters with TF-IDF.

Classifier	Precision				Recall				F1 Score			
	Neg.	Pos.	Neut.	Avg.	Neg.	Pos.	Neut.	Avg.	Neg.	Pos.	Neut.	Avg.
ADB	0.87	0.47	0.55	0.76	0.80	0.54	0.68	0.74	0.83	0.50	0.61	0.74
CC	0.92	0.50	0.64	0.81	0.83	0.65	0.74	0.79	0.87	0.57	0.69	0.80
DTC	0.80	0.41	0.50	0.68	0.77	0.42	0.55	0.67	0.78	0.42	0.52	0.67
ETC	0.94	0.41	0.56	0.82	0.79	0.64	0.74	0.77	0.86	0.50	0.64	0.78
GBM	0.89	0.40	0.55	0.76	0.79	0.53	0.66	0.73	0.83	0.46	0.60	0.74
GNB	0.26	0.32	0.84	0.61	0.92	0.28	0.24	0.37	0.41	0.30	0.37	0.36
LR	0.92	0.51	0.62	0.81	0.83	0.64	0.75	0.79	0.87	0.57	0.68	0.80
RF	0.93	0.39	0.54	0.81	0.79	0.61	0.74	0.76	0.85	0.47	0.62	0.77
SGDC	0.92	0.49	0.64	0.82	0.83	0.65	0.74	0.79	0.87	0.56	0.69	0.80
SVC	0.91	0.52	0.62	0.80	0.83	0.61	0.74	0.78	0.87	0.56	0.67	0.79
VC	0.94	0.45	0.58	0.82	0.85	0.65	0.77	0.78	0.86	0.53	0.66	0.79

**Table 13:** Classifiers’ accuracy parameters with TF feature extraction.

Classifier	Precision				Recall				F1 Score			
	Neg.	Pos.	Neut.	Avg.	Neg.	Pos.	Neut.	Avg.	Neg.	Pos.	Neut.	Avg.
ADB	0.89	0.44	0.58	0.77	0.80	0.56	0.69	0.75	0.84	0.50	0.63	0.76
CC	0.92	0.50	0.65	0.81	0.83	0.66	0.73	0.79	0.87	0.57	0.69	0.80
DTC	0.78	0.48	0.50	0.67	0.79	0.42	0.59	0.67	0.78	0.45	0.54	0.67
ETC	0.93	0.45	0.59	0.81	0.81	0.62	0.75	0.77	0.86	0.52	0.66	0.79
GBM	0.88	0.48	0.55	0.76	0.80	0.53	0.70	0.74	0.84	0.50	0.61	0.75
GNB	0.41	0.31	0.67	0.51	0.82	0.30	0.23	0.43	0.55	0.30	0.35	0.40
LR	0.90	0.57	0.65	0.80	0.85	0.62	0.73	0.79	0.87	0.59	0.69	0.79
RF	0.92	0.45	0.57	0.80	0.81	0.42	0.73	0.76	0.86	0.45	0.64	0.78
SGDC	0.88	0.60	0.67	0.79	0.86	0.60	0.71	0.78	0.87	0.60	0.69	0.78
SVC	0.86	0.59	0.66	0.77	0.86	0.58	0.70	0.77	0.86	0.58	0.68	0.77
VC	0.90	0.56	0.66	0.80	0.85	0.63	0.72	0.79	0.87	0.59	0.69	0.79

**Table 14:** Accuracy of models with word2vec feature extraction.

Classifier	Features Used	Accuracy
AdaBoost Classifier	word2vec	0.743
Calibrated Classifier	word2vec	0.780
Decision Tree Classifier	word2vec	0.623
Extra Trees Classifier	word2vec	0.737
Gaussian Naïve Bayes	word2vec	0.708
Gradient Boosting Machine	word2vec	0.715
Logistic Regression	word2vec	0.779
Random Forest Classifier	word2vec	0.746
Stochastic Gradient Descent classifier	word2vec	0.779
Support Vector Classifier	word2vec	0.783
Voting Classifier (LR + SGDC)	word2vec	0.777

**Table 15:** Comparison of classifiers’ accuracy with various feature extraction methods.

Classifier	Accuracy		
	TF	TF-IDF	Word2vec
AdaBoost Classifier	0.745	0.746	0.743
Calibrated Classifier	0.789	0.791	0.780
Decision Tree Classifier	0.672	0.686	0.623
Extra Trees Classifier	0.772	0.761	0.737
Gaussian Naïve Bayes	0.418	0.438	0.708
Gradient Boosting Machine	0.740	0.734	0.715
Logistic Regression	0.780	0.787	0.779
Random Forest Classifier	0.763	0.758	0.746
Stochastic Gradient Descent classifier	0.792	0.792	0.779
Support Vector Classifier	0.773	0.785	0.783
Voting Classifier (LR + SGDC)	0.791	0.792	0.777

**Table 16:** Accuracy of classifiers with partial pre-processing.

Classifier	Partial Pre-Processing		Complete Pre-Processing	
	Accuracy (TF)	Accuracy (TF-IDF)	Accuracy (TF)	Accuracy (TF-IDF)
AdaBoost Classifier	0.747	0.745	0.745	0.746
Calibrated Classifier	0.781	0.790	0.789	0.791
Decision Tree Classifier	0.682	0.670	0.672	0.686
Extra Trees Classifier	0.750	0.756	0.772	0.761
Gaussian Naïve Bayes	0.498	0.501	0.418	0.438
Gradient Boosting Machine	0.745	0.752	0.740	0.734
Logistic Regression	0.791	0.791	0.780	0.787
Random Forest Classifier	0.752	0.757	0.763	0.758
Stochastic Gradient Descent classifier	0.793	0.803	0.792	0.792
Support Vector Classifier	0.776	0.801	0.773	0.785
Voting Classifier (LR + SGDC)	0.794	0.804	0.791	0.792
